# BURN-SYNOVECTOMY MOUSE MODEL FOR TEMPOROMANDIBULAR JOINT OSTEOARTHRITIS

**DOI:** 10.22203/ecm.v047a04

**Published:** 2024-03-05

**Authors:** Ginny Ching-Yun Hsu

**Affiliations:** 1 Department of Orthodontics, Oregon Health & Science University, Portland, OR 97201, USA

**Keywords:** Temporomandibular joint osteoarthritis, mouse model, temporomandibular disorders

## Abstract

Temporomandibular joint osteoarthritis (TMJ OA) occurs in 8 to 16 % of the population. Currently available animal models do not faithfully simulate the native disease progression of TMJ OA. The initiation of TMJ OA requires both local trauma and extended inflammation. In this study, we present a novel mouse model that reproduces these two conditions. This is achieved by a procedure involving both synovectomy (local trauma) and a distant burn injury (systemic inflammation). Its efficacy at inducing TMJ OA was assessed by histomorphologic and radiographic evaluation at 1, 3, and 9 weeks after the procedure. Burn-synovectomy mice exhibited significantly more degenerative hard and soft tissue changes in the TMJ than uninjured control or synovotomy mice. The observed histology in burn-synovectomy mice faithfully mimicked synovitis-induced TMJ OA progression. This animal model is invaluable in future research of the mechanism and risk factors of TMJ OA.

## Introduction

Temporomandibular disorders (TMDs) affect approximately 33 % of the U.S. population (Velly *et al*., 2022). Among these, temporomandibular joint (TMJ) osteoarthritis (OA) accounts for pain symptoms in 8 to 16 % of patients (Stegenga *et al*., 1991; Toller, 1973). TMJ OA is characterized by synovitis, destruction of surrounding tissues, and erosion of the subchondral bone with different levels of pain and further results in facial asymmetry and jaw dysfunction (Wang *et al*., 2023). TMDs impact quality of life and are associated with an annual healthcare cost of ~ $4 billion in the United States alone (Bianchi *et al*., 2020).

Animal models play a crucial role in understanding the complex disease progression of TMJ OA and in evaluating new therapeutic interventions. However, lack of a reliable *in vivo* model imposes restrictions on research into the pathophysiology of TMJ OA (Xiang *et al*., 2021; Zhao *et al*., 2022). Additionally, genetically-induced animal models (Wadhwa *et al*., 2005; Kumagai *et al*., 2015) are time-consuming and expensive to construct and do not induce inflammation in the synovial capsule. Intra-articular injection is a well-characterized preclinical model which induces OA by intra-articular inflammation, cytotoxicity, and direct cartilage damage (Xiang *et al*., 2021; Zhao *et al*., 2022; Embree *et al*., 2016; Embree *et al*., 2015; Griffin *et al*., 2010; Kaul *et al*., 2016; Suchecki & Tufik, 2000; Xu *et al*., 2009). While it is easy to perform, its pathogenic mechanism differs from that of human TMJ OA, and is only suitable for the study of pain and treatment response (Haartmans *et al*., 2022; Li *et al*., 2020). Other non-invasive means of inducing TMJ OA, such as mechanical loading, high-fat diet, and sleep deprivation, only result in mild lesions, are time-consuming to construct, and may only mimic TMJ OA that arise from specific mechanisms (Griffin *et al*., 2010; Kaul *et al*., 2016; Suchecki & Tufik, 2000).

In this study, we hypothesize that the concomitant use of two procedures, namely (1) synovectomy to induce local trauma and (2) partial-thickness burn injury to induce systemic inflammation to exaggerate inflammatory events, reliably induces TMJ OA in mice in an experimental setting. Through histologic examination and microCT imaging, we seek to validate our *in vivo* animal model in its ability to mimic natural TMJ OA disease progression and its value as a future research tool.

## Materials and Methods

This is a prospective controlled animal study approved by the Institutional Animal Care and Use Committee of Oregon Health & Science University (IP00004230). All animal procedures were carried out in strict accordance with good animal practices as defined in the *Guide for the Use and Care of Laboratory Animals*.

**Fig. 1.**
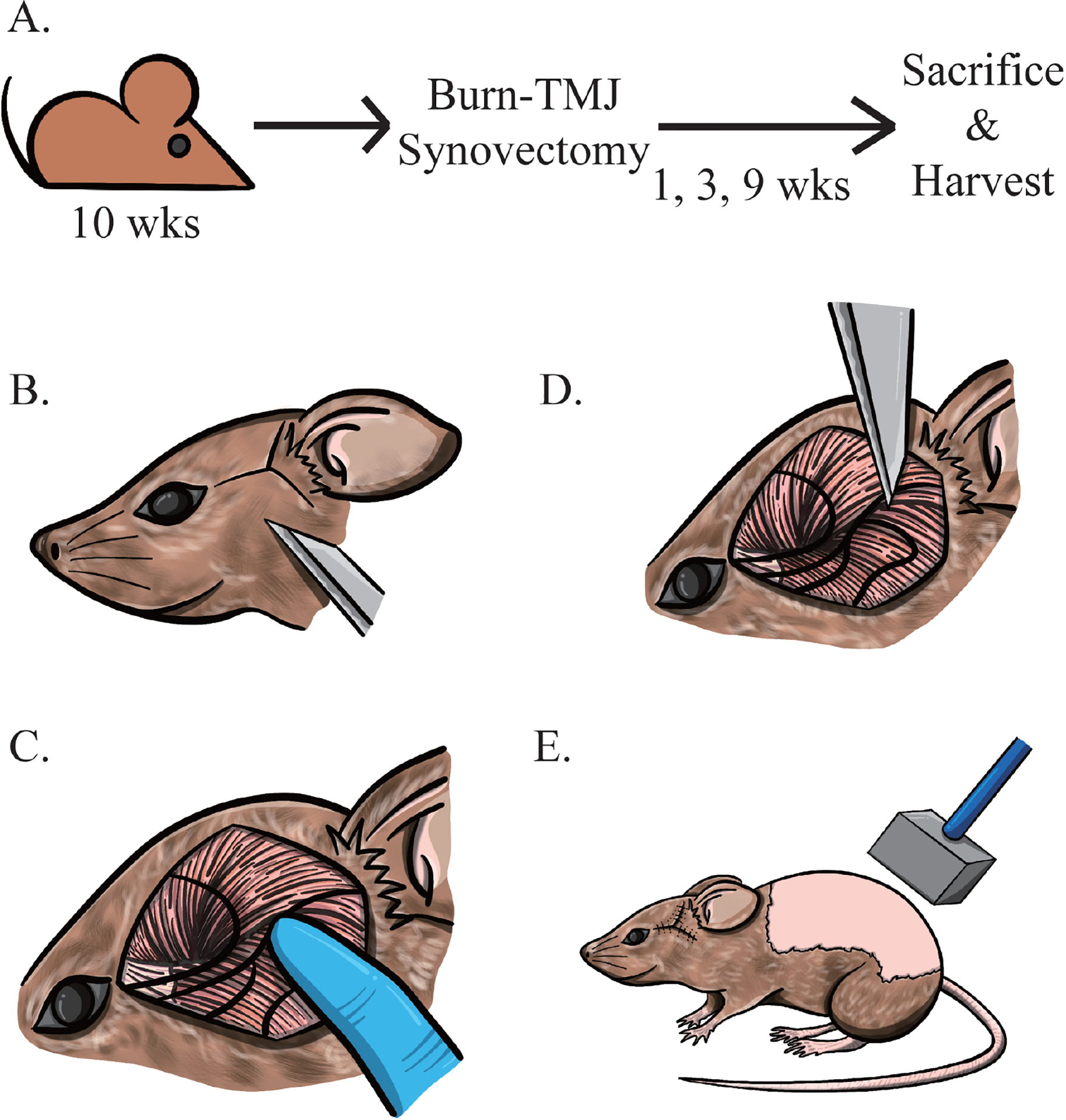
Schematic illustration of Comparative Study Design. (**A**) Experiment timeline. (**B**) Y-shaped incision to expose the zygoma extending from the lateral canthus towards the ipsilateral ear. (**C**) Identification of the TMJ at the distal zygoma by palpation. (**D**) Making a 1–2 mm incision in the synovial membrane at the posterior end of the zygoma using a #11 scalpel for 1–2 mm to make an incision and flex the joint. (**E**) Performing the partial-thickness burn on the dorsum.

A total of 54 10-week-old wild-type C57BL/6 mice were used for the current study since this animal age corresponds to the peak age when human TMJ OA occurs (Fig. 1A). Upon arrival, mice were quarantined for 2 weeks and randomly divided between three groups: (1) burn-synovectomy group (n = 6, 3 male and 3 female, in each time point), (2) synovotomy group (n = 6, 3 male and 3 female, in each time point), and (3) control group (n = 6, 3 male and 3 female, in each time point). The mice in group 1, the experimental group, underwent concomitant synovectomy with burn injury. The mice in group 2 received synovotomy alone. The mice in group 3 received no procedure.

All mice were maintained in pathogen-free, ventilated cages in the Animal facility at Oregon Health & Science University with unrestricted access to food and water in a 12 h light/dark cycle, with room temperature at 21–24 °C, humidity 55 ± 10 %. All cages contained wood shavings, bedding and nest material.

### Surgical Technique

#### Anesthesia

The animals were anesthetized using 80 mg/kg of ketamine (VETone, Boise, ID, USA) with 10 mg/kg of xylazine (Akorn Inc, Lake Forest, IL, USA) by intraperitoneal injection (IP) injection. 0.6 mg/kg of buprenorphine sustained-release (Bup SR) (Zoopharm LLC, Laramie, WY, USA) was injected subcutaneously immediately prior to the procedure.

#### Burn-synovectomy

Before synovectomy, the left cheek was shaved with clippers and disinfected with povidone-iodine. A Y-shape incision was made distal to the left eye to the left ear to expose the zygoma (Fig. 1B). The incision was extended approximately 0.5 cm to the ear canal so the TMJ could be easily visualized. The zygoma was palpated underneath the temporalis and masseter muscles until the posterior end of the zygoma was reached. The temporalis and masseter muscles were gently separated, and the TMJ distal to zygoma was identified (Fig. 1C) and marked using the tip of a #11 scalpel. The location of the TMJ was further confirmed by using the other hand to move the mandible slightly to produce condylar movement. For synovectomy mice, after the incision, the scalpel was inserted 1–2 mm from the synovial capsule to increase the mobility of the TMJ (Fig. 1D). No tissue was removed in the procedure. The skin incision was closed with 5–0 Vicryl sutures (Ethicon Inc., Raritan, NJ, USA) in a simple interrupted pattern.

During the same anesthesia session, a burn injury was made. The dorsal area from the spine to the left flank was shaved, exposing a skin area of at least 2 × 3 cm, and sterilized with povidone-iodine. A partial-thickness burn injury was inflicted using an aluminum block weighing 35 g and measuring approximately 2 × 2 × 3 cm. The aluminum block was pre-heated to 60 °C in a water bath, then applied to the shaved dorsum for 17 seconds (Fig. 1E), using gravity to hold the block in place. This created an approximately 30 % total body surface area burn in a 10-week-old mouse. Contact burn was chosen over flame or scald burn because it achieves a more uniform burn depth. The burn site was dried with gauze, and triple antibiotic ointment (Bluepoint Laboratories) which contains bacitracin, neomycin and polymyxin B was applied daily 6 days after the procedure.

#### Synovotomy

During synovotomy, a similar incision was made as described above for synovectomy, and the tip of the #11 scalpel was used to make an incision on the synovial membrane. Unlike the synovectomy that insert the scalpel into the intra-articular space to increase the mobility of the joint, synovotomy only ensure the incision on the synovial membrane. The skin was closed over the incision in a similar fashion.

#### Recovery

After the surgical procedure, mice in groups 1 and 2 received warmed fluid resuscitation using 0.5 mL Lactated Ringer’s through IP injection. The mice were given a soft diet for up to 3 days after the procedure. Subcutaneous injections of 0.6 mg/kg Bup SR (Zoopharm LLC) were administered during surgery and 72 hours post-surgery.

#### Harvest

Within each group, six animals were euthanized at 1, 3, and 9 weeks by CO_2_ inhalation followed by cervical dislocation, with their TMJ tissues harvested in accordance with institutional guidelines. These time points were selected to represent different stages in the inflammatory process.

#### Outcomes

The primary outcome is the severity of intra-articular inflammation as quantified by Osteoarthritis Research Society International (OARSI) score. Secondary outcomes include qualitative histologic examination and microCT imaging of cartilage changes. All specimens were analyzed in a blinded fashion.

After animals were sacrificed, the TMJ was collected and fixed in 4 % paraformaldehyde for 24 hours, decalcified with 14 % ethylenediaminetetraacetic acid for 14 days, and embedded in OCT compound (Sakura) (Hsu *et al*., 2020; Lee *et al*., 2021). Sagittal sections of the joint were made at a thickness of 6 μm for histology. All images were acquired using an Apotome 3 microscope (Carl Zeiss Microscopy, White Plains, NY, USA) (Lu *et al*., 2023) for qualitative histologic examination.

OARSI scoring was performed in a blinded fashion using median coronal TMJ sections to represent the weight-bearing area. Safranin O/fast green stained sections were used to assess cartilage injury. OARSI grading ranged from G1 to G6. OARSI score was calculated according to the formula: score = grade (G1 to G6) × stage (S1 to S4) (Hsu *et al*., 2021; Sono *et al*., 2020) and ranged from 0–24.

In order to quantify subchondral bone volume changes after advanced OA, we performed microcomputed tomography (μCT) on specimens harvested at week 9. We defined the region of interest for 3-dimensional reconstruction and bone volume analysis as the whole subchondral bone above the condylar neck. Using the Inveon high-resolution microcomputed tomography system (Siemens, Malvern, PA, USA), images were acquired using a 1440-step single projection pattern with 0.25 degrees arc separation. Projections were set at 80 kV and 500 μA with 610 ms exposure/projection with a 0.5-mm aluminum filter (Siemens, Saint Paul, MN, USA). The charge-coupled device magnification was set to high and the field of view was 22.56 transaxial by 8.2 mm axial. Binning was set to 0, resulting in a calculated 10.68 micron voxel size. Images were reconstructed with Inveon Acquisition Workplace software (Ver 2.1.272, Siemens, Saint Paul, MN, USA) using the Feld-kamp algorithm, no downscaling, slight noise reduction setting, Shepp-Logan filter (Siemens, Saint Paul, MN, USA), the mouse setting for beam hardening. Reconstructed images were converted to DICOM format and analyzed using ImageJ (1.53v, LOCI, University of Wisconsin, Madison, WI, USA).

### Statistical Analyses

The animal number per group were determined by power analysis. Ordinal variables are presented as median and interquartile ranges and analyzed using the two-way Mann-Whitney-U test. Continuous variables are presented as mean ± standard deviation and analyzed using two-way ANOVA followed by Tukey’s post hoc tests. Differences with *p* < 0.05 were considered statistically significant. All statistical analyses are performed using commercial software (GraphPad Prism 9.5, GraphPad Software, Inc. San Diego, CA, USA).

## Results

We were able to execute the procedures and specimen harvest described above with no deaths outside of planned euthanasia time points. Quantitative data from the three experimental groups are shown in Table 1.

### Histologic Examination

**Fig. 2.**
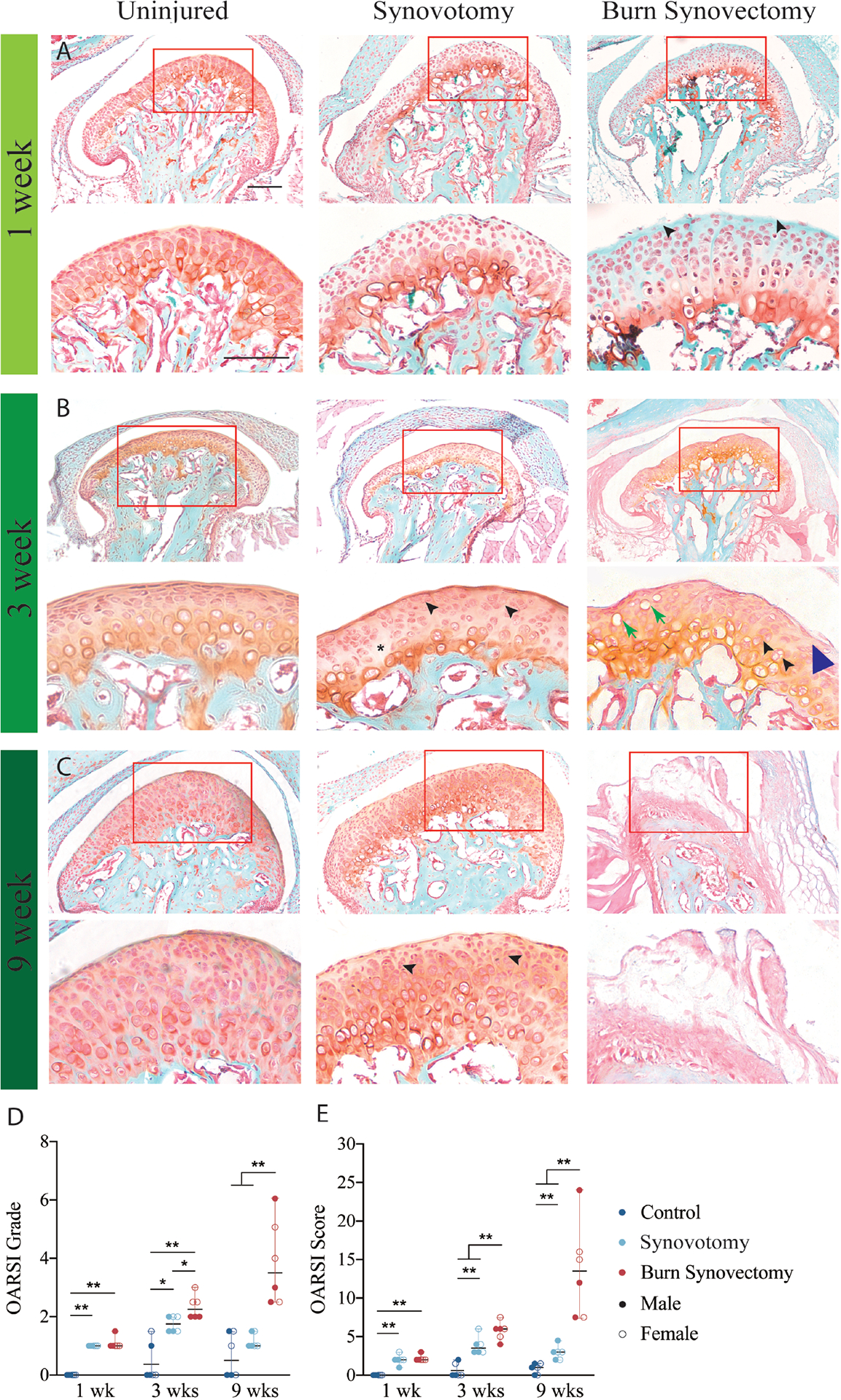
Histologic Examination of TMJ Sections. (**A-C**) Histology of the TMJ sections stained with Fast Green and Safranin O Red and histopathology grade assessment. Uninjured (left column), synovotomy (middle column) and burn-synovectomy (right column) mice were sacrificed after (**A**) 1 week, (**B**) 3 weeks, or (**C**) 9 weeks and the TMJ was harvested. Within each row, low- (upper panels) and high (lower panels)-magnification images of the condyle morphology are shown. *, acellular areas under the articular surfaces. Blue arrowheads, discontinuities in the condylar surface. Black arrowhead, loss of regular columnar chondrocyte structure. Green arrow, apoptotic chondrocytes. (**D,E**) Osteoarthritis Research Society International (OARSI) grades (**D**) and scores (**E**) were evaluated at each timepoint. Data are expressed as means, with dots representing individual animals. n = 6 (3 male and 3 female) per timepoint per condition. *, *p* < 0.05, **, *p* < 0.01 within the same time point. Groups with different letters indicate significant differences (*p* < 0.05) between different time points. Scale bars = 200 μm (low magnification), 50 μm (high magnification).

We compared the severity of TMJ OA between burn-synovectomy mice and mice that received only synovotomy or no injury by histologic examination (Fig. 2). While the control mice remained normal, both burn-synovectomy and synovotomy mice exhibited histologic features of OA in the condyle as early as 1 week after surgery. These include multifocal decreases in cell count (asterisks) and the formation of chondrocyte clusters (black arrows) within the superficial zone (Fig. 2A). At 3 weeks, burn-synovectomy mice exhibited more severe OA than synovotomy mice and uninjured control mice, as evidenced by their more irregular superficial zone (blue arrow), increased hypertrophic and apoptotic chondrocytes (green arrow), and increased chondrocyte clusters (black arrow) from the superficial to zone of chondrocytes (Fig. 2B). The synovotomy mice also exhibited increased lesions compared to the uninjured control mice. However, the majority of the lesions appeared at the superficial layer of the condyle. At 9 weeks, the degenerative changes continued to progress, resulting in calcified bone erosion and cartilage fissures (Fig. 2C).

We used the OARSI system to quantify these degenerative changes (Fig. 2D,E), scoring the degree of inflammation based on cell morphology, cartilage integrity and subchondral bone involvement. At week 1, the burn-synovectomy group and synovotomy group displayed similar levels of increased lesions compared to the uninjured controls (Fig. 2D,E), signifying an intact surface with minimal chondrocyte clusters involving less than 25 % of the examined area. At week 3, the lesions in the burn-synovectomy group were significantly increased compared to the synovotomy and uninjured control groups in grade and score, and the synovotomy group also exhibited a significant increase in pathological lesions compared to the uninjured controls. Interestingly, the burn-synovectomy group exhibited significantly increased lesions compared to the synovotomy group in grade but not in score. At week 9, condylar destruction became even more pronounced in the burn-synovectomy group, with full depth erosion of fibrocartilage (Fig. 2C). The synovotomy group had significantly increased score but not grade compared to the uninjured group.

### Morphological Analyses of TMJ OA

**Fig. 3.**
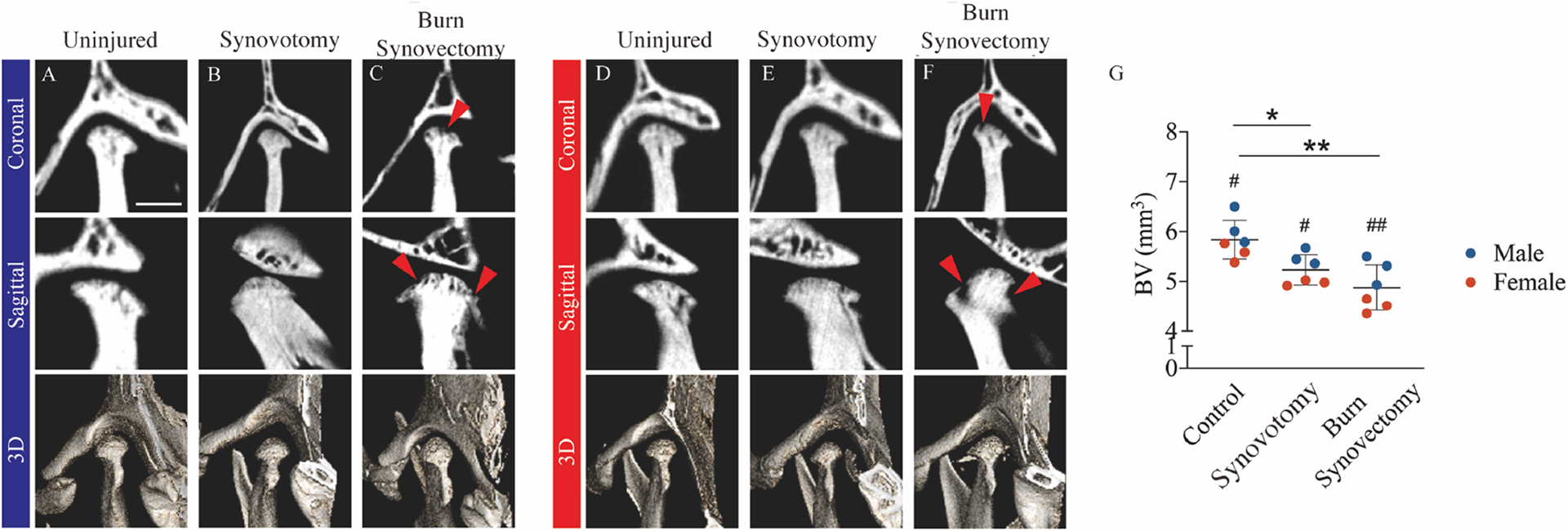
Radiographic changes in condylar subchondral bone. Micro-computed tomography (μCT) of the left TMJ of male (**A-C**) and female (**D-F**) animals at 9 weeks post-injury. Representative images of coronal (upper), sagittal (middle) views and 3D reconstructions (lower) of left TMJs with uninjured control (**A,D**), synovotomy (**B,E**) and burn-synovectomy (**C,F**). Red arrows, bone erosion on the surface of the condyle. (**G**) μCT quantification of condyle bone volume. *, *p* < 0.05, **, *p* < 0.01 between different conditions and #, *p* < 0.05, ##, *p* < 0.01 between sexes within the same condition. Scale bar = 1 mm.

2D micro-computed tomography (μCT) images of specimens harvested at week 9 were obtained for all three comparison groups (Fig. 3). In the control group, normal joint morphology with intact and uniform condylar subchondral bone was found (Fig. 3A). While the synovotomy group displayed only limited subchondral changes (Fig. 3B), the burn-synovectomy mice displayed severe defects on the condylar head (red arrow) (Fig. 3C). Similarly, on 3D image reconstruction, control and synovotomy mice had smoother bone morphology, while craters were visible in the burn-synovectomy group.

Severity of bone loss was quantified using bone volume analysis. Significantly higher bone volume was found in the synovotomy and control groups than the burn-synovectomy group. Quantitative analysis revealed significantly decreased bone volume in the burn-synovectomy group and synovotomy group compared to the uninjured controls (Fig. 3G). All three groups were evaluated between sexes. Male mice generally had increased bone volume compared to their female counterparts, but the differences were increased in the burn-synovectomy group.

## Discussion

In the present study, an *in vivo* animal model that reliably simulates native TMJ OA disease progression was developed. OA was induced by concomitant synovectomy, which causes local traumatic injury, and a partial-thickness burn injury, which causes systemic inflammation. Local trauma combined with pro-inflammation factors is believed to be the initiator for TMJ OA degenerative changes. Our histologic and radiologic results showed that mice that received the burn-synovectomy procedure had more cartilage tissue reaction, increased erosion, and subchondral bone turnover compared to synovotomy and uninjured mice, indicating that mice receiving burn-synovectomy exhibited more salient features of TMJ OA degeneration than mice receiving either synovotomy alone or no injury.

This is the first experimental animal model with a mechanism that imitates native TMJ OA. Due to the variability and difficulty of obtaining TMJ samples from human patients, animal models play a key role for TMJ OA research (Xiang *et al*., 2021; Li *et al*., 2020). Our current study shows that a burn-synovectomy animal model faithfully replicates the sequence of cellular events by using systemic inflammation to exacerbate and expedite the local inflammation by local synovectomy. During the progression of burn-synovectomy induced TMJ OA, starting from articular cartilage degeneration and progressing to the over-production of proteoglycans and other extracellular matrix molecules and appearance of hyperplastic and proliferative chondrocyte clusters (Xu *et al*., 2009). Then, as proteoglycans decrease on the articular cartilage surface, the fibrocartilage layer fissures. This degenerative sequence of events eventually affects the subchondral calcified bone, leading to a loss of bone volume, causing high bone turnover and aggressive osteoblast and osteoclast activity (Karsdal *et al*., 2008; Pastoureau *et al*., 2010). During the TMJ OA degenerative changes, we also observed a more significant difference in bone volume between male and female mice in burn-synovectomy group compared to synovotomy and control groups, which mimics the sex differences in TMJ OA patients, as female TMJ OA patients are twice more than male patients.

Another strength of our model is its consistency. We observed similar severity and distribution of inflammation in between animals at each harvest time point. Our procedures facilitate standardization, as long as sterile technique are observed, and attention was paid towards uniform contact between the aluminum block and mouse skin. The pain control by sustained-release buprenorphine was administrated during healing and lasted for 6 days after surgery procedure so no significant abnormal mouse behavior was observed throughout the process. One limitation to our animal model lies in its translatability to human conditions. There is limited evidence in the literature to support the notion that systemic inflammation arising from distant burn sites increases the risk of TMJ OA. While TMJ OA is frequently observed in patients with craniofacial or cervical burn, it is unclear whether trunk or limb burn patients develop TMJ OA at a higher incidence (Hilbert *et al*., 1984; Rubin & Cozzi, 1986; Schwartz *et al*., 1976). In contrast, patients with connective tissue disorders and autoimmune diseases such as rheumatoid arthritis and systemic lupus erythematosus have been reported to have higher incidence rates of TMJ OA degeneration due to the widespread inflammation. More importantly, increased studies have supported the notion that local derangement with subclinical inflammation/extended inflammation are the initiators of TMJ OA. Our burn-synovectomy mouse model has demonstrated that local injury is not enough to cause TMJ degenerative changes, which require aggressive or extended inflammation. However, it is unclear if the systemic inflammation induced by burn injury is comparable to those induced by other conditions, such as autoimmune diseases and LPS-induced infection with regard to its effect on the TMJ (Ananias *et al*., 2023; Juan *et al*., 2023; Seemann *et al*., 2017). Further analyses are needed to evaluate differences in TMJ inflammation from local injury only compared to systemic inflammation or if behavior/psychological changes induced by inflammation are in the TMJ OA progression.

To our knowledge, this is the first study that successfully simulates primary TMJ OA using a combined burn and synovectomy model, incorporating the effects of systemic inflammation to exaggerate the local inflammation by local trauma. Due to its reproducibility and ease of operation, this model is ideal for research of the disease pathophysiology and treatment response.

## Figures and Tables

**Table 1.** Summary of Radiographic and Histologic Findings on Various Groups.

	Burn-synovectomy (n = 18)	Synovotomy (n = 18)	Uninjured control (n = 18)
**Harvested at week 1 (n = 6 in each group)**			
**OARSI grade (mean +/− SD)**	1.083 ± 0.204	1.0 ± 0	0 ± 0
**OARSI score (mean +/− SD)**	2.167 ± 0.408	2.0 ± 0.632	0 ± 0
**Harvested at Week 3 (n = 6 in each group)**			
**OARSI grade (mean +/− SD)**	2.333 ± 0.408	1.75 ± 0.274	0.417 ± 0.665
**OARSI score (mean +/− SD)**	5.75 ± 1.173	3.833 ± 1.169	0.583 ± 0.917
**Harvested at week 9 (n = 6 in each group)**			
**OARSI grade (mean +/− SD)**	3.833 ± 1.438	1.167 ± 0.204	0.667 ± 0.753
**OARSI score (mean +/− SD)**	13.667 ± 6.210	2.917 ± 0.917	0.833 ± 0.683
**Bone volume (mm** ^**2**^ **, mean +/− SD)**	4.878 ± 0.457	5.235 ± 0.304	5.839 ± 0.386

OARSI, Osteoarthritis Research Society International.

## List of Abbreviations

TMJ: Temporomandibular join
OA: osteoarthritis
TMDs: Temporomandibular disorders
IP: intraperitoneal injection
OARSI: Osteoarthritis Research Society International
